# High Loss to Followup and Early Mortality Create Substantial Reduction in Patient Retention at Antiretroviral Treatment Program in North-West Ethiopia

**DOI:** 10.5402/2012/721720

**Published:** 2012-06-14

**Authors:** Mamo Wubshet, Yemane Berhane, Alemayehu Worku, Yigzaw Kebede, Ermias Diro

**Affiliations:** ^1^Institute of Public Health, University of Gondar, P.O. Box 196, Gondar, Ethiopia; ^2^Addis Continental Institute of Public Health, P.O. Box 26751/1000, Addis Ababa, Ethiopia

## Abstract

*Background*. There has been a rapid scale up of antiretroviral therapy (ART) in Ethiopia since 2005. We aimed to evaluate mortality, loss to followup, and retention in care at HIV Clinic, University of Gondar Hospital, north-west Ethiopia. *Method*. A retrospective patient chart record analysis was performed on adult AIDS patients enrolled in the treatment program starting from 1 March 2005. We performed survival analysis to determine, mortality, loss to followup and retention in care. *Results*. A total of 3012 AIDS patients were enrolled in the ART Program between March 2005 and August 2010. At the end of the 66 months of the program initiation, 61.4% of the patients were retained on treatment, 10.4% died, and 31.4% were lost to followup. Fifty-six percent of the deaths and 46% of those lost to followup occurred in the first year of treatment. Male gender (adjusted hazard ratio (AHR) was 3.26; 95% CI: 2.19–4.88); CD4 count ≤200 cells/**μ**L (AHR 5.02; 95% CI: 2.03–12.39), tuberculosis (AHR 2.91; 95% CI: 2.11–4.02); bed-ridden functional status (AHR 12.88; 95% CI: 8.19–20.26) were predictors of mortality, whereas only CD4 count <200 cells/**μ**L (HR = 1.33; 95% CI: (0.95, 1.88) and ambulatory functional status (HR = 1.65; 95% CI: (1.22, 2.23) were significantly associated with LTF. *Conclusion*. Loss to followup and mortality in the first year following enrollment remain a challenge for retention of patients in care. Strengthening patient monitoring can improve patient retention AIDS care.

## 1. Background

Ethiopia is one of the few countries with the highest number of people living with HIV/AIDS globally. According to Ethiopian Demographic and Health Survey (EDHS) 2011, Federal Ministry of Health (FMOH) and HIV/AIDS Prevention and Control Office (HAPCO) estimated that adult HIV prevalence was 1.5% of which 73,000 people require ART 2010 [1]. A fee-based ART program in 2003, and a free antiretroviral therapy (ART) program in early 2005 was started [2]. Subsequently, a number of initiatives have been undertaken to expand the availability of ART in Ethiopia. Task shifting and decentralization of the service to increasing numbers of both health centers and hospitals was done since August 2006 [3].

The provision of antiretroviral treatment has decreased morbidity and mortality in people living with HIV [3–7]. There have been several enabling factors for rapid scale-up of ART in resource-limited settings. Despite recent progress in improving access to ART, limited uptake, poor retention, and difficulties in accessing care remain a serious concern for ART programs [3, 4, 8–10].

 Treatment discontinuation raises some of the concern about drug resistance, which incomplete adherence does, and negates much of the benefit sought by those implementing treatment programs [11, 12]. Patients with clinical AIDS who discontinue ART will likely die within a relatively short time [13]. Long-term retention of patients in treatment programs has received far less attention perhaps because most large-scale treatment providers have few resources available to track missing patients [4, 10]. As a result, much attention has focused on patient day-to-day adherence to antiretroviral medications [10].

There are also concerns regarding high rates of lost to followup and early mortality in a number of sub-Saharan African ART programs [14–16]. High rates of attrition from treatment programs thus pose a serious challenge to program implementers and constitute an inefficient use of scarce treatment resources [17, 18]. Attrition from these antiretroviral treatment programs is mainly due to the death and loss to followup of the patient [4, 8, 19]. There is thus a legitimate concern that a focus on numbers of people initiated on ART may compromise quality of care [4].

The feasibility of implementing ART in resource limited countries has been demonstrated in multiple studies, with one-year outcomes often comparable to those in developed countries [4, 5, 19–21]. Little is known about the longer-term sustainability of such outcomes in such programs. The aim of this study is, therefore, to describe the ART program practices and clinical outcome as measured by attrition (mortality and loss to followup) and retention care at the University of Gondar (UoG) Hospital, ART Clinic, from March 2005 to August 2010. Examination of these data helps to understand the challenges that must be overcome to ensure the long-term success of treatment services in the treatment program in Ethiopia.

## 2. Method

### 2.1. Study Design and Setting

A retrospective cohort analysis was conducted at the UoG Hospital, ART Clinic, north-west Ethiopia. Unpublished report by UoG showed that the hospital is a leading referral hospital in north-west Ethiopia serving an estimated of five million people. Summary of medical records of the hospital showed that as of January, 2012, about 9,019 patients were enrolled at the AIDS care clinic, out of which 3, 516 adult clients started on ART in the clinic. As part of the government program, the clinic provides care and support to people living with HIV free of charge using the national and WHO guidelines [2, 22]. The ART clinic has begun using electronic database in 2006 to monitor the performance of ART program. This database has been used to provide aggregate data on key indicators. Patients at the ART clinic are referred by ART clinician to case managers for adherence and managing ART risk factors. Case managers are trained high school graduates with modest experience of community health practice. They are required to conduct planned outreach activities to trace those lost to follow-up and onsite community support [23]. After ART initiation, patients were given monthly review appointments [2].

### 2.2. Inclusion Criteria and Definitions

All adult, nonpregnant, AIDS patients in the treatment program from March 2005 through August 2010 were eligible. Patients were excluded if ART initiation or termination date was missing, and/or if dates were wrongly recorded such as, ART initiation date after ART termination date. Patients who failed to return on their designated appointments date were traced by case managers. Initial tracing was attempted by telephone by the clinic nurses on the day following the missed appointment. If this was unsuccessful, case managers were deployed by their respective addresses. Care was taken not to disclose HIV status to families or other household members. Patients who could not be traced and did not subsequently return to the clinic during the study period were classified as lost to followup (LTF). Data about death was collected from patents' charts. Any death confirmed and recorded in the patient card on ART was assumed to be related to HIV/AIDS, unless and otherwise specified. Patients who notified clinic staff of their intention to transfer to another facility were given a written referral form and classified as transfer out (TO). Retention to care was defined as those patients under the study known to be alive and receiving ART regularly at UoG Hospital ART Clinic as of August 30, 2010. Attrition was defined as discontinuation of ART for reasons related to death or loss to followup. Transfers to another health facility, when reported, were not regarded as attrition because these patients who transferred were assumed to be retained in another facility. However, actual retention to the facility was considered transfer-outs as the patient were assumed to be no more a part of the ART program.

### 2.3. Measurement and Statistical Analysis

Data were retrieved from patients' ART card by trained nurses working in the ART Clinic using uniform data abstraction format prepared for the study. Whenever relevant information was missing, the ART database was consulted. Outcome measures were death, LTF, and retention to care. Length of followup varied because of different enrollment time. Observation was censored at the date of LTF or transfer out or at the end of the study period, 30 August 2010. Time to death or LTF was censored the date of the last clinic visit.

Mortality and LTF overtime were evaluated by declaring data at each calendar year, considering time of entry and exit to determine the contribution of time spent in the cohort. Every year entry and exit observations were used to calculate person-time, event counts, and incidence rates at each calendar times. The overall rate was calculated in the same fashion at the end of the follow-up time. Then mortality and lost to follow-up rates were calculated by dividing deaths or LTF by person-year at each calendar time to get incidence rates per 100 person-years of observation.

We estimated time to death and LTF and retention to care by Kaplan-Meier failure and survival functions, respectively. To get insight into the shape of the survival function for each group and give an idea of whether or not the groups were proportional, we used the Kaplan-Meier curves for all the categorical predictors. We tested equality across strata to explore whether or not to include the predictor in the final model. For the categorical variables, we used the log-rank test of equality across strata. For the continuous variables, we used the Cox proportional hazard model with a single continuous predictor. We considered including the predictor if the test had a *P* value of 0.20 or less. For our model building, we considered the model which included all the predictors that had a *P* value of less than 0.20 in the univariate analyses. Covariates predetermined to be in the univariate analysis were entered into full multivariable Cox proportional hazard model. The following baseline covariates were entered into the model: age, gender, marital status, occupation, disclosure status, type of initial regimen, the presence of tuberculosis, place of residence, functional status, WHO stage, and CD4 count. Trend overtime was tested by Mantel Haenszel Chi-square for liner trend.

This study obtained ethical clearance from the Institutional Review Board of the University of Gondar, Ethiopia. No personal identifiers were declared in the data set prepared for analysis and in the study report.

## 3. Result

### 3.1. Patient Baseline Characteristics

**Figure 1:**
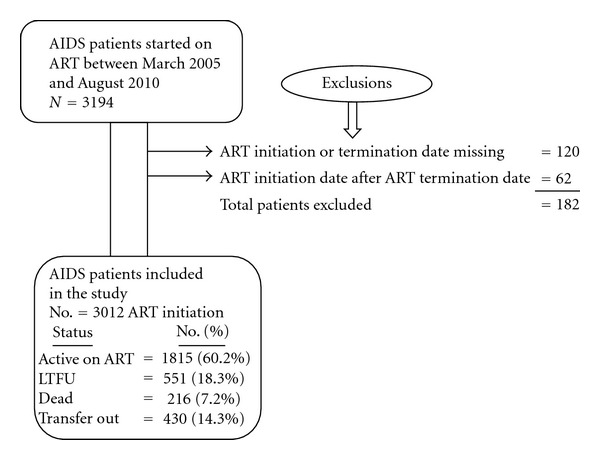
Schematic Diagram Showing the ART Program at UoG hospital, AIDS care, from March 2005 to August 2010.

Between March 2005 and August 2010, 3194 patients were initiated on ART, out of which 3012 were considered in the analysis (Figure 1). The total follow-up time was 6,386.2 person-years. Sociodemographic and clinical baseline patient characteristics of the 3012 AIDS patients who were on ART is presented on Tables 1 and 2. The median age was 32 years (IQR 27–40); 55.2% were female, and 45% of the patients were separated, divorced, or widowed. Majority of the patients were Christian by religion (92.4%), and came from urban areas (96.1%). Thirty-five percent and 54% of the patients were uneducated and unemployed, respectively. When patients initially presented, 75% disclosed their HIV status to one or more person they knew. Eligibility for ART was determined by CD4 count in 55.3%, and both by CD4 and clinically in 39% of the patients. The median baseline CD4 count was 120 cells/*μ*L (IQR 63–195). Baseline CD4 count in 42% of the patients was less than 200 cells/*μ*L. The majority of the patients (84%) were presented with WHO stages III and IV. The most common type of initial regimen used was Stavudine, Lamuvudine, and Neverapine (36%) or Efaveranze (19%). Twenty-seven percent of the cases were presented with tuberculosis coinfected with HIV.

### 3.2. Retention of Patients on ART

During 6,386.2 patient-years of followup, 216 (7.2%) died, 551 (18.4%) LTF, 430 (14.3%) transferred to other health facility, and 1815 (60.3%) were retained in the program. Survival analysis with Kaplan Meier estimates of retention into care was 87.5% (95 CI: 86.3–88.7) at 6 month, 80.7% (95 CI: 79.2–82.1) at 12 months, 73.8% (95 CI: 72.1–75.5) at 24 months, 69.3% (95 CI: 67.2–71.2) at 48 months, and 61.4% (95 CI: 47.3–72.7) at the end of the follow-up period (5.5 years). The probability of surviving 5.5 years was more than 50%. Attrition was mainly due to LTF (31.4%, 95% CI: 19.5–48.1) followed by death (10.4%, 95% CI: 9.0–12.1). The majority of the deaths and lost to follow-ups occurred at 6 and 12 months of followup. Fifty-six percent of deaths and 46% of lost to follow-ups occurred in the first year of treatment (Table 3).

### 3.3. Mortality and LTF Rates

As shown in Table 4, the overall incidence of mortality and LTF rates were 3.4 per 100 person-years (95% CI: 3.0–3.9), and 8.4 per 100 person-years (95% CI: 7.8–9.2), respectively. Mortality and LTF were highest in the first 3 months, 8.3 per 100 person-years (95% CI: 6.5–10.7) and 18.7 per 100 person-years (95% CI: 15.8–22.2), respectively; from ART initiation to 6 months, these rates were 7.7 per 100 person-years (95% CI: 6.4–9.4) and 18.8 per 100 person-years (95% CI: 16.7–21.4), respectively; between 6 and 12 months after ART initiation, these rates declined to 4.0 per 100 person-years (95% CI: 2.9–5.3) and 12.1 per 100 person-years (95% CI: 10.3–14.4), respectively.

**Figure 2:**
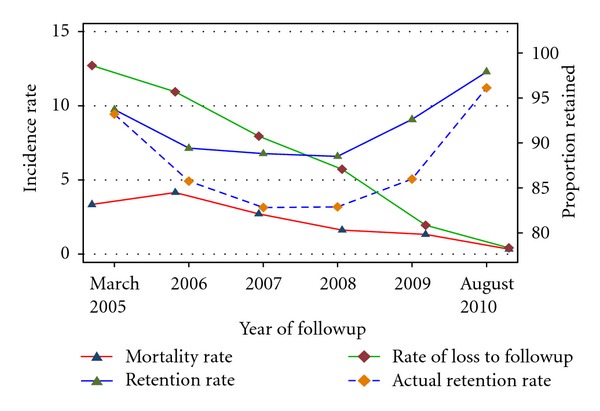
Trends of mortality, lost to followup, and retention by calendar year at university of Gondar Hospital ART Program, between March 2005 and August 2010.

Trend of mortality, LTF and retention in care is presented in Figure 2 and Table 3. The rate of mortality declined from 3.3 per 100 person-years (95% CI: 1.4–8.0) in 2005 to 0.3 per 100 person-years (95% CI: 0.2, 0.5) in 2010. This decline in the incidence of mortality was statistically significant (Test for trend, *P* < 0.02). Likewise the rate of LTF declined from 12.7 per 100 person-years (95% CI: 8.1, 19.9) in 2005 to 0.41 (95% CI: 0.3–0.6) in 2010 (Test for trend, *P* < 0.001).

The incidence of mortality for patients with CD4 cell count <50 cells/*μ*L increased from 7.4 per 100 person-years (95% CI: 5.9–9.3) from the baseline to 12.9 per 100 person-years (95% CI: 8.2–20.3) after 24 months of followup. However, in patients with CD4 cell count >200 cells/*μ*L, mortality decreased from 2.7 per 100 person-years (95% CI: 2.1–3.4) at the baseline to 2.3 per 100 person-years (95% CI: 1.6–3.0) after 24 months of followup.

**Figure 3:**
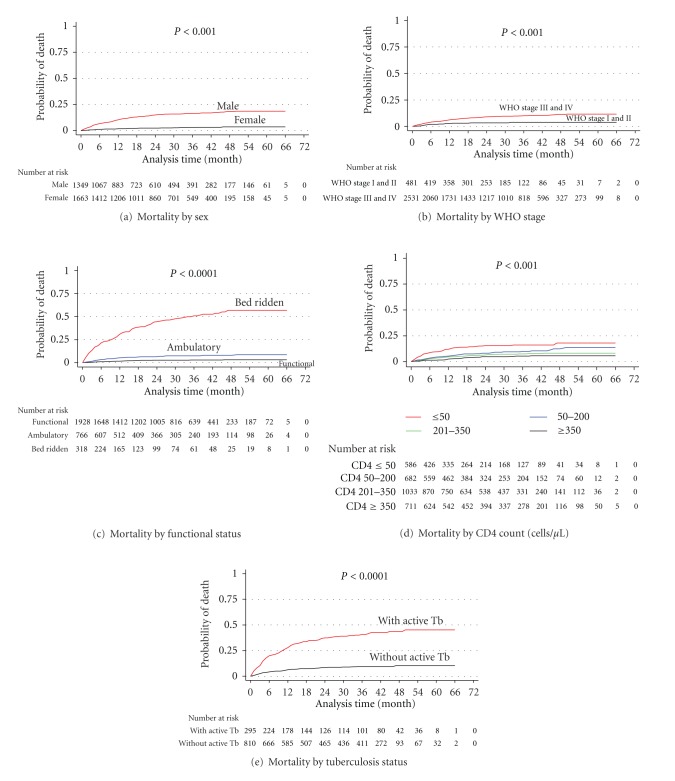
Kaplan-Meier Estimate of Mortality by baseline characteristics among AIDS patients on ART at University of Gondar Hospital ART Program, between March 2005 and August 2010.

Cox proportional hazards model demonstrated that being male (HR = 3.26; 95% CI: (2.19, 4.88), ambulatory and bedridden functional status (HR = 2.20; 95% CI: (1.30, 3.70) and (HR = 12.88; 95% CI: (8.19, 20.26), resp.). CD4 count <200 cells/*μ*L (HR = 5.02; 95% CI: (2.03, 12.39), and the presence of HIV-tuberculosis coinfection at ART initiation (HR = 2.91; 95% CI: (2.11, 4.02) were significantly associated with mortality. In contrast, CD4 count <200 cells/*μ*L (HR = 1.33; 95% CI: (0.95, 1.88) and ambulatory functional status (HR = 1.65; 95% CI: (1.22, 2.23) were significantly associated with LTF. As shown in Figure 3, WHO stages III and IV were significantly associated with mortality in bivariate analysis (HR = 2.6 with 95% CI: (1.6, 4.5); when adjusted for other variables, the direction of the relationship changed and turned out insignificant, which resulted from the small number of deaths occurring at WHO stages I and II (Table 5).

## 4. Discussion

This study adds to the limited number of researches on ART outcome assessments from sub-Saharan Africa regarding concerns related to high rates of LTF, early mortality, and challenges of retention of AIDS patients in long-term care [8, 16, 20, 21, 24, 25]. The analysis of 3012 patients initiating ART in 5.5 years in a referral teaching hospital in north-west Ethiopia ART Program demonstrated that more than 60% of patients initiated on ART were retained. LTF was the major reason for patient exit from the cohort. In addition the study shows that the majority of the deaths and LTF occurred in the first 3 months of followup. This study thus provides longer assessment of AIDS clinic outcomes over those reported from similar studies, which reported outcomes of up to two years [4, 15, 19].

Patient retention is a vital measure of the effectiveness of ART services [26]. Retention in long-term care is complex, especially in low- and middle-income countries [8, 25, 27]. The findings of this study document the difficulties in retaining patients in care for life-long treatment due to LTF. Patient retention in care in this program (61.4%) is comparable to the findings of other sub-Saharan countries [4, 15]. For example, Assefa et al., in the study of outcomes of the ART services in the 55 health facilities in Ethiopia, found out that ART programs in Ethiopia are able to retain on average 68% (95% CI: 51–85) of their patients in 2 years [4]. Likewise, a recent systematic review in sub-Saharan Africa ART programs by Fox and Rosen (2010) demonstrated that these programs were able to retain about 60% of their patients at the end of 2 years [8]. The ART program in this hospital, therefore, has a better picture of the effectiveness of ART delivery at 5.5 years as compared to previous studies, which suggested that less than 50% of the patients were retained at a shorter follow-up time [20, 28]. The probability of surviving 5.5 years is more than 50% and hence the chance of surviving to the last followup was high. This program, therefore, has better effectiveness in retention of patients in care after 5.5 years, suggesting a better success, although there is no clear threshold at which retention in care is considered adequate.

Mortality is one reason for patient attrition in ART programs. We observed a low overall mortality rate of 3.4 per 100 person-years, comparable to studies done in Africa [9]. There was also relatively lower death rate of 7.7 per 100 person-years in the first 6 months of ART compared to other similar ART Clinics [14, 16, 29]. For example, the death rate observed in Arbaminch hospital in Ethiopia demonstrated an overall mortality rate of 9.1 per 100 person-years [16]. The low mortality rates observed in our cohort may, in part, be due to access to tertiary care level diagnostics and hospital care. Another possibility is that some of those LTF might have died. As a program expands, its ability to accurately ascertain patient deaths deteriorates, and high observed LTF may be associated with poor mortality ascertainment [14, 30].

Nonetheless, mortality is not regarded as a major reason for patient attrition in large ART programs in developing countries. The greater threat to the success of many African ART program is the observation of high levels of LTF, insofar as this outcome reflects patients who have truly left care [24]. LTF increases throughout the follow-up period resulting in decline in retention of care. Rate of LTF of 8.4 per 100 person-years in our cohort is comparable to that observed in the South Africa studies [9, 16], but relatively higher as compared to the rate observed in AIDS Clinical Trial Group (ACTG) analysis, which is 5.1 per 100 person-years of observation [31]. The size and pace of ART scale-up may have contributed to the observed LTF. The program has grown in size dramatically, with our cohort increasing enrolment about 8-fold from 2005 to 2010. In contrast to these individuals, patients who are truly LTF are likely to be nonadherent to treatment and at higher risk of death [31, 32]. In addition, they face increased risk of drug resistance to ART, undermining the long-term effectiveness of treatment programs [9, 33].

The finding of this study should be interpreted with caution and has got a number of limitations. To start with, death was ascertained by patient record review. Consequently, deaths which occurred at home might have not been documented in the patient card. This may underestimate the rate of mortality. Besides, patients' card was the main source of information in our study. In the data collection, however, we learned that some of the data lack accuracy and completeness. This may underestimate or overestimate some outcome of interest. Therefore, health care facilities must ensure the quality of patients' records. Second, there were a high number of patients who were LTF or transferred to another ART site following the country-wide roll-out of ART in our study. Though we assumed that those transfered out were retained in another health facility, our assumption may not hold true as these patients might not be in care. The actual retention in our study considering this scenario of transfer out as a means of attrition reduces retention to the level of 46%. Besides, some LTF might have died, although we have little reason to believe these misclassifications would be anything more than random.

Lastly, the study is institutional based and more than 96% of the study cohort was from urban residence that might be able to reach to get the service than the rural people. This may overestimate retention, though residence was not a predictor for both mortality and LTF in our cohort. As a result, the generalizability of the study may be limited. Future research should be targeted to population level and rural areas of the country.

In conclusion, this study highlights that patient retention in chronic AIDS care remains a challenge during scalingup of the program. Though overall mortality rate has declined, attrition from the program is mainly due to increase in rates of LTF and early mortality. Close patient monitoring, particularly soon after the initiation of therapy, is important. Facilities must set up simple, standardized monitoring system to track the numbers of patients starting therapy every quarter and determine the treatment outcomes. Further research is needed to better understand the reasons of early mortality. In addition, future research should also be directed towards understanding the contextual reasons and outcomes of patients LTF and transferred to other treatment facilitates. This may stimulate programmatic improvements aimed at retention to care. This information could also be potentially useful in the development of strategies designed to increase patient followup and long-term retention in treatment programs.

## Figures and Tables

**Table 1:** Baseline Sociodemographic profile of AIDS patients on ART at University of Gondar Hospital ART program between March 2005 and August 2010.

Variable	Frequency (%)
Gender	
Male	1,349 (44.8)
Female	1,663 (55.2)
Age (years)	
15–24	330 (11.0)
25–34	1,332 (44.2)
35–44	954 (31.7)
≥45	396 (13.65)
Marital status	
Married	1,041 (34.6)
Single	613 (20.4)
Separated, divorced, and widowed	1,358 (45.1)
Religion	
Muslim	218 (7.2)
Christian	2,784 (92.4)
Others	10 (0.3)
Education	
No education	1,061 (35.2)
Primary	858 (28.5)
Secondary	761 (25.3)
Tertiary	332 (11.0)
Occupation	
Employed	1,388 (46.1)
Unemployed	1,624 (53.9)

**Table 2:** Baseline clinical characteristics of AIDS patients on ART at University of Gondar Hospital ART program, between March 2005 and August 2010.

Variable	Frequency (%)
HIV disclosure status	
Disclosed	2260 (75.0)
Not disclosed	752 (25.0)
*Initial ART regimen	
d4T-3TC-NVP	1086 (36.1)
d4T-3TC-EFV	559 (18.6)
d4T-3TC-NVP	916 (30.4)
d4T-3TC-EFV	421 (14.0)
Others	30 (1.0)
Initial regimen changed?	
yes	1537 (75.5)
no	498 (24.5)
Baseline WHO stage	
Stage I	109 (3.6)
Stage II	372 (12.4)
Stage III	1980 (65.7)
Stage iv	551 (18.3)
Baseline functional status	
Functional	1928 (64.0)
Ambulatory	766 (25.4)
Bed ridden	318 (10.6)
Tuberculosis present	
yes	295 (26.7)
No	810 (73.3)
Baseline CD4 cell count (cells/*μ*L)	
<50	586 (19.5)
50–200	682 (22.6)
201–350	1033 (34.3)
>350	711 (23.6)

*d4T: Stavudine; 3TC: Lamivudine; NVP: Nevirapine; EFV: Efavirenz; AZT: Zidouvudine; TDF: Tenofovir disoproxil fumarate.

**Table 3:** Kaplan-Meier estimate of mortality, loss to followup, and retention by baseline characteristics among AIDS patients on ART at University of Gondar Hospital ART program between March 2005 and August 2010.

Duration of followup	*n *	Mortality % (95% CI)	LTF % (95% CI)	Retention % (95% CI)
6 months	2479	3.7 (3.1–4.5)	9.0 (8.0–10.1)	87.5 (86.3–88.7)
12 months	2089	5.6 (4.8–6.6)	14.4 (13.1–15.8)	80.7 (79.2–82.1)
18 months	1734	7.1 (6.1–8.2)	17.7 (16.3–19.2)	76.4 (74.7–78.0)
24 months	1470	8.1 (7.0–9.3)	19.6 (18.1–21.2)	73.8 (72.1–75.5)
36 months	940	9.1 (7.9–10.4)	21.5 (19.9–23.3)	71.3 (69.4–73.0)
48 months	372	10.2 (8.8–11.6)	22.8 (21.1–24.7)	69.3 (67.2–71.2)
60 months	106	10.4 (9.0–12.1)	23.8 (21.9–25.9)	68.2 (66.0–70.3)
66 months	10	10.4 (9.0–12.1)	31.4 (19.5–48.1)	61.4 (47.3–72.7)

**Table 4:** Mortality, loss to followup, and retention by calendar year at university of Gondar Hospital ART program, between March 2005 and August 2010.

Cohort of patients on ART	Patients representing the cohort (a)		^§^Death		^§^Lost to followup		^§^Attrition no. (%)	^§^Retention (%) no. (%)	^§^Transfer out no. (%)	^§^Actual retention to the facility no. (%)
		Person-year (b)	No. of deaths (c)	Rate per 100 PYO (95% CI)	No. LTF (e)	Rate per 100 PYO (95% CI)				
				^¥^(**d**) = **c**/**b**		^¥^(**f**) = **e**/**b**	^¥^(**f**) = (**c** + **e**)/**a**	^¥^(**g**) = (1 − **f**)/**a**		^¥^(**i**) = (**g** − **h**)/**b**
2005	383	149.5	5	3.3 (1.4–8.0)	19	12.7 (8.1–19.9)	24 (6.3)	359 (93.7)	2 (0.5)	357 (93.21)
2006	1024	721.3	30	4.2 (2.9–5.9)	79	11.0 (8.8–13.7)	109 (10.6)	915 (89.4)	37 (3.6)	878 (85.74)
2007	1727	1809.1	49	2.7 (2.0–3.6)	144	8.0 (6.8–9.4)	193 (11.2)	1534 (88.8)	104 (6.0)	1430 (82.80)
2008	2047	3217.8	52	1.6 (1.2–2.1)	184	5.7 (4.9–6.6)	236 (11.5)	1811 (88.5)	114 (5.6)	1697 (82.90)
2009	2075	4659.3	62	1.3 (1.0–1.7)	91	2.0 (1.6–2.4)	153 (7.4)	1922 (92.6)	138 (6.7)	1784 (85.97)
2010	1876	5354.1	18	0.3 (0.2–0.5)	22	0.4 (0.3–0.6)	40 (2.1)	1916 (97.2)	33 (1.8)	1791 (95.47)

Overall	3012	6383.2	216	3.4 (3.0–3.9)	^†^539	8.4 (7.8–9.2)	755 (25.5)	1815 (60.3)	430 (14.3)	1385 (46.0)

^**§**^Death—when confirmed and recorded in the patient card; Lost to followup—if more than three months late for a scheduled visit, and if the tracing team was unable to get information; Transfer-outs—patients who got written referral form to another facility; Attrition—discontinuation of ART for reasons related to death and loss to follow-up; Retention to care—those patients known to be alive and receiving ART; Actual retention—patients actually retained in the program that do not consider transfer out. ^¥^Incidence mortality and loss to followup are calculated by dividing events at each cohort by the corresponding PYO. Attrition rate is numbers of death plus LTF divided by patients representing the cohort. Retention is calculated total patients representing the cohort minus attrition divided by total patients representing the cohort. ^†^ART—Antiretroviral Therapy; UoG—University of Gondar; PYO-Person—years of observation; LTF—lost to followup.

**Table 5:** Cox-proportional Hazard Model of association between baseline characteristics and death and loss to followup among AIDS patients on ART at University of Gondar Hospital ART Program, from March 2005 to August 2010.

Baseline characteristics	Outcome death	Outcome LTFU
	AHR (95% CI)	AHR (95% CI)
Gender		
Male	3.26 (2.19–4.88)	0.95 (0.72–1.25)
Female	1.00	1.00
WHO stage		
Stage I or II	1.00	0.86 (0.53–1.40)
Stage III or IV	0.68 (0.34–1.16)	1.00
CD4 count (cells/*μ*L)		
<200	5.02 (2.03–12.39)	1.33 (0.95–1.88)
≥200	1.00	
Functional status		
Functional	1.00	1.00
Ambulatory	2.20 (1.30–3.70)	1.65 (1.22–2.23)
Bed ridden	12.88 (8.19–20.26)	0.87 (0.52–1.43)
Tuberculosis at ART initiation		
Yes	2.91 (2.11–4.02)	0.88 (0.63–1.22)
No	1.00	1.00

## References

[1] Central Statistics Agency (CSA) (2011). Ethiopian demographic and health survey. *Preliminary Report*.

[2] Federal Ministry of Health (2007). Antiretroviral treatment program implementation guideline in Ethiopi.

[3] Assefa Y, Jerene D, Lulseged S, Ooms G, Van Damme W (2009). Rapid scale-up of antiretroviral treatment in Ethiopia: successes and system-wide effects. *PLoS Medicine*.

[4] Assefa Y, Kiflie A, Tesfaye D (2011). Outcomes of antiretroviral treatment program in Ethiopia: retention of patients in care is a major challenge and varies across health facilities. *BMC Health Services Research*.

[5] Boulle A, Van Cutsem G, Hilderbrand K (2010). Seven-year experience of a primary care antiretroviral treatment programme in Khayelitsha, South Africa. *AIDS*.

[6] Reniers G, Araya T, Davey G (2009). Steep declines in population-level AIDS mortality following the introduction of antiretroviral therapy in Addis Ababa, Ethiopia. *AIDS*.

[7] Corey DM, Kim HW, Salazar R (2007). Brief report: effectiveness of combination antiretroviral therapy on survival and opportunistic infections in a developing world setting: an observational cohort study. *Journal of Acquired Immune Deficiency Syndromes*.

[8] Fox MP, Rosen S (2010). Patient retention in antiretroviral therapy programs up to three years on treatment in sub-Saharan Africa, 2007–2009: systematic review. *Tropical Medicine and International Health*.

[9] Sanne IM, Westreich D, Macphail AP, Rubel D, Majuba P, van Rie A (2009). Long term outcomes of antiretroviral therapy in a large HIV/AIDS care clinic in urban South Africa: a prospective cohort study. *Journal of the International AIDS Society*.

[10] Barnighausen T, Chaiyachati K, Chimbindi N, Peoples A, Haberer J, Newell ML (2011). Interventions to increase antiretroviral adherence in sub-Saharan Africa: a systematic review of evaluation studies. *The Lancet Infectious Diseases*.

[11] Van Damme W, Kober K, Laga M (2006). The real challenges for scaling up ART in sub-Saharan Africa. *AIDS*.

[12] Unge C, Södergård B, Marrone G (2010). Long-term adherence to antiretroviral treatment and program drop-out in a high-risk urban setting in sub-Saharan Africa: a prospective cohort study. *PLoS ONE*.

[13] Keiser O, Tweya H, Braitstein P (2010). Mortality after failure of antiretroviral therapy in sub-Saharan Africa. *Tropical Medicine & International Health*.

[14] Brinkhof MWG, Dabis F, Myer L (2008). Early loss of HIV-infected patients on potent antiretroviral therapy programmes in lower-income countries. *Bulletin of the World Health Organization*.

[15] MacPherson P, Moshabela M, Martinson N, Pronyk P (2009). Mortality and loss to follow-up among HAART initiators in rural South Africa. *Transactions of the Royal Society of Tropical Medicine and Hygiene*.

[16] Mulissa Z, Jerene D, Lindtjørn B (2010). Patients present earlier and survival has improved, but pre-ART attrition is high in a six-year HIV cohort data from Ethiopia. *PLoS ONE*.

[17] Rosen S, Fox MP (2011). Retention in HIV care between testing and treatment in sub-Saharan Africa: a systematic review. *PLoS Medicine*.

[18] Ferradini L, Jeannin A, Pinoges L (2006). Scaling up of highly active antiretroviral therapy in a rural district of Malawi: an effectiveness assessment. *The Lancet*.

[19] Cohen R, Lynch S, Bygrave H (2009). Antiretroviral treatment outcomes from a nurse-driven, community-supported HIV/AIDS treatment programme in rural Lesotho: observational cohort assessment at two years. *Journal of the International AIDS Society*.

[20] Auld AF, Mbofana F, Shiraishi RW (2011). Four-year treatment outcomes of adult patients enrolled in Mozambique’s rapidly expanding antiretroviral therapy program. *PLoS ONE*.

[21] Bussmann H, Wester CW, Ndwapi N (2008). Five-year outcomes of initial patients treated in Botswana’s National Antiretroviral Treatment Program. *AIDS*.

[22] WHO/UNAIDS (2006). patient monitoring guidelines for HIV care and antiretroviral therapy.

[23] Marseille EA, Kevany S, Ahmed I (2011). Case management to improve adherence for HIV-infected patients receiving antiretroviral therapy in Ethiopia: a micro-costing study. *Cost Effectiveness and Resource Allocation*.

[24] Cornell M, Grimsrud A, Fairall L (2010). Temporal changes in programme outcomes among adult patients initiating antiretroviral therapy across South Africa, 2002–2007. *AIDS*.

[25] Geng EH, Hunt PW, Diero LO (2011). Trends in the clinical characteristics of HIV-infected patients initiating antiretroviral therapy in Kenya, Uganda and Tanzania between 2002 and 2009. *Journal of the International AIDS Society*.

[26] Boyles TH, Wilkinson LS, Leisegang R, Maartens G (2011). Factors influencing retention in care after starting antiretroviral therapy in a rural south african programme. *PLoS ONE*.

[27] Toure S, Kouadio B, Seyler C (2008). Rapid scaling-up of antiretroviral therapy in 10,000 adults in Côte d’Ivoire: 2-year outcomes and determinants. *AIDS*.

[28] Rosen S, Fox MP, Gill CJ (2007). Patient retention in antiretroviral therapy programs in sub-Saharan Africa: a systematic review. *PLoS Medicine*.

[29] Jerene D, Endale A, Hailu Y, Lindtjøorn B (2006). Predictors of early death in a cohort of Ethiopian patients treated with HAART. *BMC Infectious Diseases*.

[30] Brinkhof MWG, Spycher BD, Yiannoutsos C (2010). Adjusting mortality for loss to follow-up: analysis of five art programmes in sub-saharan africa. *PLoS ONE*.

[31] Krishnan S, Wu K, Smurzynski M (2011). Incidence rate of and factors associated with loss to follow-up in a longitudinal cohort of antiretroviral-treated HIV-infected persons: an AIDS Clinical Trials Group (ACTG) Longitudinal Linked Randomized Trials (ALLRT) analysis. *HIV/AIDS Clinical Trials*.

[32] Grimsrud A, Ford N, Myer L (2011). Defaulting from antiretroviral treatment programmes in sub-Saharan Africa: a problem of definition. *Tropical Medicine and International Health*.

[33] Zhang F, Dou Z, Ma Y (2009). Five-year outcomes of the China National Free Antiretroviral Treatment Program. *Annals of Internal Medicine*.

